# Estimating price and expenditure elasticities for select foods and drinks in South Africa using a demand systems model

**DOI:** 10.1177/22799036251350956

**Published:** 2025-06-29

**Authors:** Chengetai Dare, Maxime Bercholz, Micheal Kofi Boachie, Evelyn Thsehla, Shu Wen Ng

**Affiliations:** 1 SAMRC/WITS Centre for Health Economics and Decision Science - PRICELESS SA, Wits School of Public Health, Faculty of Health Sciences, University of the Witwatersrand, Johannesburg, South Africa; 2 Carolina Population Center, University of North Carolina at Chapel Hill, NC, USA; 3 Department of Nutrition, University of North Carolina at Chapel Hill, NC, USA

**Keywords:** demand model, price elasticity, income, South Africa, health economics

## Abstract

**Background:**

South Africa implemented a Health Promotion Levy (HPL) on sugar- sweetened beverages in 2018 and has a draft regulation on front-of-package labeling for packaged foods containing excess sugar, sodium, or saturated fats. Estimates on price elasticities of demand for these products do not exist to date; the implications of expanding the HPL are thus unknown.

**Design and methods:**

We employ a modified exact affine Stone index demand system model to estimate the expenditure and uncompensated own- and cross-price elasticities of demand for select foods and beverages in South Africa using purchase data from lower- and higher-SES households from January 2016 through March 2019.

**Results:**

We found own-price elasticities of demand ranging from −1.05 for packaged fruits, vegetables, nuts, and seeds (FVNS) to −1.91 for low-sugar dairy drinks, implying that a 10% rise in prices reduces the demand for these commodities by 10.5%–19.1%. Lower-SES South African households are generally more price sensitive. Some goods are substitutes (e.g. 100% fruit juice and other soft drinks) while others (e.g. desserts and FVNS) are weak complements.

**Conclusions:**

The government may have room to raise and expand the HPL to further discourage consumption of these products and raise additional revenue, although the total effect would also depend on supply side responses, which we are unable to capture here.

## Significance for public health

There is limited knowledge about the responsiveness of ultra-processed foods and sugary drinks to changes in prices (and health taxes) in South Africa. This study estimated total expenditure and uncompensated own- and cross-price elasticities of demand for selected food and beverage groups, by socioeconomic level, using novel and detailed food, and beverage purchase data among South African households. This is particularly important in the South African context where economic inequalities persist, and the consequences of public policy are not always clear. Findings from this study lay the foundation for future work to simulate changes in purchases by socio-economic group under different fiscal policy scenarios including taxes and subsidies, and from that determine the population and subpopulation-specific nutritional implications and estimate the potential tax revenues that might be generated.

## Introduction

South Africa faces a growing burden of overweight and obesity, with 31% of adult males, 67% of adult females, and 13% of children under five classified as overweight or obese in 2016.^1,2^ Without intervention, projections estimate that 50% of South Africans will be obese by 2030.^3^ These conditions are major risk factors for non-communicable diseases (NCDs), including type 2 diabetes, hypertension, and cardiovascular diseases, posing significant challenges to the health system and economy.^4,5^ The consumption of ultra-processed foods (UPFs) and sugar-sweetened beverages (SSBs), which are energy- dense and high in sugar, saturated fats, and sodium, is a key driver of this epidemic. In South Africa, UPFs accounted for nearly 40% of energy intake among low-income households in 2017–2018.^6^

To address this public health crisis, the National Department of Health implemented the Health Promotion Levy (HPL) on SSBs in April 2018, set at 0.021 ZAR per gram of sugar above 4 g per 100 ml, constituting an approximate 10% tax on popular SSBs.^7^ Proposed regulations, such as front-of-package labeling for packaged foods high in sugar, sodium, or saturated fats, aim to further curb unhealthy consumption.^8,9^ Socioeconomic disparities exacerbate the obesity and NCD burden, with lower-income households facing higher exposure to affordable, nutrient-poor UPFs and limited access to healthier alternatives.^6^ These communities bear a disproportionate share of NCD-related morbidity and mortality, deepening health inequities.^10^ Fiscal policies like the HPL, if expanded, could shift consumption patterns, particularly among price-sensitive lower-income groups, while generating revenue for health promotion.^7^

Despite these interventions, the effectiveness of expanding the HPL to other UPFs or adjusting its rate remains unclear due to limited evidence on price and expenditure elasticities for these products in South Africa. This study aims to estimate own- and cross-price elasticities of demand for selected food and beverage groups, stratified by socioeconomic status, to inform potential fiscal policy expansions, and their health and revenue implications.

### Literature review

The global rise in overweight and obesity, particularly in sub-Saharan Africa (SSA), has been well-documented, with prevalence rates in SSA increasing from 9% to 23% for men and 17% to 39% for women between 1990 and 2022.^11^ In South Africa, obesity rates are among the highest in the region, with significant economic costs estimated at US$7.6 billion (2% of GDP) in 2019, projected to rise to US$42 billion (4.8% of GDP) by 2060.^12^ Overweight and obesity are major risk factors for non-communicable diseases (NCDs), including type 2 diabetes, hypertension, and cardiovascular diseases.^4,5^

A substantial body of evidence links the rise in NCDs to increased consumption of ultra- processed foods (UPFs), defined as energy-dense, nutritionally unbalanced products high in sugar, saturated fats, and sodium.^4,5,13,14^ UPFs, such as SSBs, packaged snacks, and processed meats, contribute significantly to dietary intakes globally, accounting for 50%–60% of energy intake in high-income countries and nearly 40% among low-income South Africans in 2017–2018.^6,15,16^ UPFs displace healthier, minimally processed foods, leading to nutritional, social, and economic externalities.^17,18^

In response to rising obesity, South Africa implemented the Health Promotion Levy (HPL) on SSBs in 2018, set at 0.021 ZAR per gram of sugar above 4 g per 100 ml, resulting in an approximate 10% tax.^7^ Studies evaluating the HPL found a 32% reduction in SSB purchases among lower Living Standard Measure (LSM) households and a 27% reduction among higher LSM households, with sugar reductions (51%) exceeding volume reductions (29%) due to reformulation.^7,19^ However, supply-side responses, such as similar price increases for low- and high-sugar beverages, may have diluted the HPL’s impact.^19^ (LSM is a market research tool developed by the South Africa Audience Research Foundation to stratify South Africa’s population into 10 socioeconomic groups based on household characteristics and access to goods and services).

Existing price elasticity estimates for South Africa are limited to SSBs, alcohol, and tobacco, with no specific focus on UPFs.^18–20^ Estimates from an Almost Ideal Demand System (AIDS) model include elasticities of −1.37 for soft drinks, −1.18 for sweet snacks, and −1.12 for desserts.^20^ In contrast, evidence from Chile suggests UPFs are highly price- elastic, with elasticities exceeding one.^21^ Previous South African studies using broader food categories reported elasticities of −1.78 for dairy and −0.99 for fruits and vegetables, aligning with findings from more disaggregated data.^20,22^ However, there remains a critical gap in understanding the price responsiveness of UPFs and other sugary beverages in South Africa, particularly by socioeconomic group, which is essential for evaluating the potential health and fiscal impacts of HPL expansion.

## Methods

We estimated total expenditure and uncompensated (Marshallian) own- and cross-price elasticities of demand for eight beverage groups, four food groups, and all other food and beverage items (numeraire) using a modified exact affine Stone index (EASI) demand system model,^23^ conditional on total food and beverage expenditure for at-home consumption. Importantly, unlike an AIDS model, an EASI demand system allows for any rank and its Engel curves can be represented by polynomials of any order, providing more flexibility to capture non-linear spending patterns across income levels. The specification allows for the addition of new demographic or socioeconomic characteristics as explanatory variables in the demand equations (as shifters), potentially interacting with price and expenditure terms to capture more nuanced effects.

For prices, we constructed household-quarter Fisher price indices for the food and beverage groups (excluding the numeraire), with group-brand elements and base prices and quantities set at their sample means. We imputed missing unit price values due to non-purchasing using predictions from group-level regressions of observed unit price values. Demographic demand shifters included household size, family life cycle (empty nesters, families, mature without children, retired, and young without children), socio-economic status (lower and higher LSM), and province and quarter fixed effects. We estimated all equations excluding the numeraire separately by maximum likelihood and derived estimates of the parameters and variance-covariance matrix for the EASI system of equations. We then computed the compensated price and expenditure elasticities and their standard errors from the estimated EASI model parameters. Details may be found in Appendix A of the Supplemental Materials.

### Data

We used data on household food and beverage purchases from Kantar’s South African consumer panel on fast-moving consumer goods (FMCGs) from January 2016 through March 2019. Even though this period includes observations prior to the announcement and implementation of the HPL and covers a year after the implementation of the HPL, we have no reason to believe that the HPL would meaningfully shift price responsiveness across food and beverage groups. Moreover, the longer period allows for greater price variability in the data and a larger sample size to allow for stratified analyses by socioeconomic level. While this dataset is somewhat dated, it is the best available dataset for this study at the time of writing and is well within the range of other studies of consumer demand using similar datasets.^24–26^ Furthermore, to our knowledge, this is the first study to estimate an EASI demand system model using household scanner data for South Africa, or any other African country more widely.

The Kantar sample consists of approximately 3000 households in any given month and covers both urban and rural areas across all provinces and most of the socioeconomic spectrum. Because South Africa’s poorest households are less likely to have electricity, which is required to report purchases, the sample excludes the lowest three LSM groups—an estimated 4% of the total household population in 2018.^7^ The Institutional Review Board at the University of North Carolina at Chapel Hill did not consider this study of secondary, deidentified data to be human-subjects research.

Panel members were asked to report all their food and beverage purchases taken home using a handheld scanner and a barcode booklet for items without barcodes. Demographics were collected upon recruitment and updated annually. Households reporting purchases in less than five categories or fewer than one shopping trip per week were replaced by demographically similar prospective panelists throughout the study period. These strict reporting criteria led to a high turnover and an unbalanced panel, resulting in the final dataset including quarterly observations from the median household for five out of the 13 quarters assessed.

Using nutrient values from other sources previously matched to the beverage purchases^7^ and Kantar’s food categories, we classified purchases into eight beverage groups, four food groups, and a numeraire. To that end, we dropped beverages that were not matched to the nutrient data or otherwise could not be classified because of missing sugar content. We also excluded non-numeraire foods with an integer package size less than 10 g or 10 ml (possibly incorrect size or unit of measure), household-month-items outside the numeraire with a unit value lying more than five standard deviations from the item’s group mean (splitting groups by unit of measure where applicable), Northern Cape due to its small number of households, and a small number of observations with a household size less than or equal to the number of children. These exclusions yielded 28,697 household- quarter observations from 4704 households, with 2158 lower-LSM and 2546 higher- LSM households.

Table 1 describes the weighted sample by LSM and overall. Compared to population estimates from the General Household Survey for lower LSM households,^7^ the weighted sample has a noticeably greater proportion of households from LSM groups 6–9 (69%–72% vs 55%–58%), and smaller proportion from groups 4 to 5 (23%–26% vs 38%–39%). The average sample household has slightly fewer children (0.9 vs 1.1), consistent with a younger household head (40–41 vs 46). Lastly, the distribution of the sample across provinces is similar to that of the population of LSM 4–10 households, with slightly greater and smaller proportions from Limpopo (12%–13% vs 9%) and Eastern Cape (7%–8% vs 10%), in addition to the exclusion of Northern Cape (0% vs 2%).

**Table 1.** Weighted sample descriptives.

Variable	Lower LSM	Higher LSM	All
Living standards measure			
4	14%	0%	8%
5	26%	0%	15%
6	60%	0%	36%
7	0%	32%	13%
8	0%	26%	11%
9	0%	28%	11%
10	0%	13%	5%
Family life cycle			
Empty nesters	10%	16%	13%
Families	55%	44%	51%
Mature without children	14%	19%	16%
Retired	2%	5%	3%
Young without children	19%	16%	17%
Household head’s age			
18–39	61%	48%	56%
40–59	32%	41%	36%
60+	7%	11%	8%
Mean	38.2	42.5	40.0
Household size			
1	19%	21%	20%
2	19%	20%	19%
3	17%	16%	17%
4	17%	18%	17%
5+	28%	24%	27%
Mean	3.4	3.2	3.3
No. of children			
0	45%	56%	49%
1	28%	23%	26%
2	17%	17%	17%
3+	10%	5%	8%
Mean	1.0	0.7	0.9
Province			
Western Cape	4%	24%	12%
Eastern Cape	8%	8%	8%
Free State	10%	5%	8%
Kwa-Zulu Natal	19%	14%	17%
North-West	8%	3%	6%
Gauteng	24%	41%	31%
Mpumalanga	8%	3%	6%
Limpopo	19%	3%	13%
No. of obs.	13,030	15,667	28,697
No. of households	2158	2546	4704
Median no. of obs. per household	4	5	5

### Groups

Beverages were classified according to beverage type, and sugar content (up to vs over 4 g of sugar per 100 ml) if subject to the HPL, with some exceptions. Non-taxable flavored water and coffee and tea drinks with over 4 g of sugar per 100 ml were excluded from bottled water and coffee and tea, respectively, and included in the numeraire. Conversely, non-taxable soft drinks and dairy drinks with over 4 g of sugar per 100 ml were included in high-sugar soft drinks and high-sugar dairy drinks and alternatives, respectively. Each of these beverage types represent under 0.5% of total beverage volume purchased. Although this study focused on beverages, we also included four food groups of interest.

Three being discretionary food groups (chocolate and candy; desserts; snacks) and one healthy food group (fruit, vegetables, nuts, and seeds). Other food groups were included in the numeraire, alongside other beverages. Table 2 reports the weighted mean budget shares overall and conditional on a purchase, together with the proportion of non- purchasers, for the full sample, and by LSM. Group expenditures and quantities per person are described in Supplemental Appendix B Table 1.

**Table 2.** Weighted mean budget shares (%).

	Lower LSM			Higher LSM			All		
Food or Beverage Category	*w*	(*w* = 0)	*w*|*w* > 0	*w*	(*w* = 0)	*w*|*w* > 0	*w*	(*w* = 0)	*w*|*w* > 0
Bottled water	0.4	76.2	1.7	0.4	70.6	1.5	0.4	74.0	1.6
100% juice	1.2	64.8	3.3	1.3	51.8	2.8	1.2	59.6	3.0
Low-sugar (LS) soft drinks	0.7	74.3	2.7	0.8	66.4	2.3	0.7	71.1	2.5
High-sugar (HS) soft drinks	7.3	14.6	8.6	5.3	15.5	6.2	6.5	14.9	7.7
Coffee and tea	3.8	28.3	5.3	3.7	26.6	5.0	3.7	27.6	5.2
Milk and LS dairy drinks and alternatives	4.4	23.8	5.8	3.5	23.4	4.6	4.0	23.6	5.3
HS dairy drinks and alternatives	0.6	77.8	2.5	0.7	72.4	2.4	0.6	75.6	2.5
Alcoholic beverages	5.4	67.5	16.6	4.5	67.0	13.6	5.0	67.3	15.4
Chocolate and candy	1.4	52.3	3.0	2.8	30.1	4.1	2.0	43.3	3.5
Desserts	3.1	30.5	4.4	3.5	20.1	4.3	3.2	26.3	4.4
Snacks	2.8	27.9	3.9	3.2	17.9	4.0	3.0	23.9	3.9
Fruit, vegetables, nuts, and seeds (FVNS)	5.1	20.1	6.3	6.1	11.4	6.9	5.5	16.6	6.6
Numeraire	63.9	0.5	64.2	64.2	0.4	64.4	64.0	0.5	64.3

*Note.* “*w*” denotes budget share (%) among all households; “*w* = 0” denotes share of households that did not report purchasing any item within the group, and “*w*|*w* > 0” denotes budget share (%) only among households that reported purchasing an item within the group. LS and HS are defined as up to and over 4 g of sugar per 100 ml, respectively. Bottled water includes LS flavored water. LS and HS soft drinks include carbonated soft drinks, juice drinks and fruit-flavored drinks. Snacks include sweet and salty varieties. All other foods and beverages (packaged and non-packaged) are included in the numeraire.

## Results

Table 3 presents the price elasticity estimates for the selected food groups for the full sample. The results show own-price elasticities above 1 in absolute value, ranging from −1.05 for packaged fruits, vegetables, nuts, and seeds (FVNS) to −1.91 for low-sugar dairy drinks, indicating that demand for these groups is price-elastic. Specifically, the own-price elasticity of −1.54 for bottled water indicates that a 10% increase in the retail price of bottled water will be associated with a 15.4% decline in the demand for bottled water. In the case of 100% fruit juice and soft drinks (both low- and high-sugar), the decline in demand is 15.5%, 19.1% and 12%, respectively, for every 10% increase in price. For alcoholic drinks, the own-price elasticity is −1.43 (Supplemental Appendix B Table 2).

**Table 3.** Average price (*e_i,_*) and expenditure (*e_i_*) elasticities, full sample.

	*ei,j*						
Food or Beverage Category	*j* = 1	*j* = 2	*j* = 3	*j* = 4	*j* = 5	*j* = 6	*j* = 7
1. Bottled water	−1.54*** (0.10)	0.12 (0.07)	0.15** (0.05)	0.13 (0.07)	−0.02 (0.06)	0.01 (0.07)	0.09 (0.07)
2. 100% juice	0.07 (0.04)	−1.55*** (0.08)	0.03 (0.04)	0.14** (0.05)	0.01 (0.04)	0.03 (0.05)	0.08 (0.05)
3. Low-sugar (LS) soft drinks	0.12** (0.04)	0.04 (0.05)	−1.91*** (0.07)	0.17** (0.05)	0.01 (0.05)	−0.02 (0.06)	0.07 (0.05)
4. High-sugar (HS) soft drinks	0.04* (0.02)	0.06** (0.02)	0.06*** (0.02)	−1.20*** (0.03)	0.03 (0.02)	0.01 (0.02)	0.02 (0.02)
5. Coffee and tea	−0.00 (0.02)	0.01 (0.02)	0.01 (0.02)	0.04 (0.03)	−1.35*** (0.03)	0.02 (0.03)	0.04 (0.03)
6. Milk and LS dairy drinks and alternatives	0.01 (0.02)	0.03 (0.03)	−0.00 (0.03)	0.01 (0.03)	0.02 (0.03)	−1.25*** (0.03)	0.01 (0.03)
7. HS dairy drinks and alternatives	0.07 (0.06)	0.10 (0.06)	0.07 (0.05)	0.04 (0.06)	0.08 (0.06)	0.02 (0.06)	−1.67*** (0.09)
8. Alcoholic beverages	−0.02** (0.01)	−0.01 (0.01)	−0.01 (0.01)	−0.05*** (0.01)	−0.02 (0.01)	−0.02* (0.01)	0.01 (0.01)
9. Chocolate and candy	0.11** (0.04)	0.05 (0.04)	0.01 (0.04)	0.06 (0.04)	0.05 (0.03)	−0.05 (0.03)	0.01 (0.04)
10. Desserts	0.02 (0.02)	−0.01 (0.03)	0.01 (0.02)	−0.08* (0.03)	0.08*** (0.02)	−0.01 (0.02)	0.01 (0.02)
11. Snacks	0.01 (0.03)	0.07* (0.03)	−0.02 (0.02)	−0.03 (0.03)	0.05 (0.02)	0.01 (0.03)	0.06* (0.03)
12. Fruit, vegetables, nuts and seeds	0.00 (0.02)	0.02 (0.02)	−0.01 (0.02)	−0.02 (0.02)	0.00 (0.02)	0.00 (0.02)	−0.03 (0.02)
13. Numeraire	−0.00 (0.00)	0.00 (0.00)	0.01*** (0.00)	0.01** (0.00)	0.01* (0.00)	0.02*** (0.00)	0.00 (0.00)
	*ei,j*						
	*j* = 8	*j* = 9	*j* = 10	*j* = 11	*j* = 12	*j* = 13	*e_i_*
1. Bottled water	−0.16** (0.05)	0.21** (0.07)	0.03 (0.06)	0.02 (0.07)	−0.01 (0.07)	−0.43* (0.17)	1.28*** (0.02)
2. 100% juice	−0.00 (0.04)	0.05 (0.05)	−0.02 (0.04)	0.08 (0.04)	0.02 (0.04)	−0.30* (0.13)	1.25*** (0.01)
3. LS soft drinks	−0.02 (0.05)	0.01 (0.05)	0.02 (0.05)	−0.04 (0.04)	−0.04 (0.05)	0.17 (0.15)	1.31*** (0.02)
4. HS soft drinks	−0.02 (0.02)	0.03 (0.02)	−0.04* (0.02)	−0.02 (0.02)	−0.01 (0.02)	0.10 (0.05)	0.95*** (0.01)
5. Coffee and tea	0.02 (0.02)	0.03 (0.02)	0.07*** (0.02)	0.03 (0.02)	−0.00 (0.02)	−0.02 (0.07)	1.06*** (0.01)
6. Milk and LS dairy drinks and alternatives	0.01 (0.02)	−0.03 (0.02)	−0.00 (0.02)	0.01 (0.02)	0.00 (0.03)	0.19* (0.07)	0.99*** (0.01)
7. HS dairy drinks and alternatives	0.06 (0.06)	0.01 (0.06)	0.01 (0.05)	0.08 (0.05)	−0.09 (0.05)	−0.22 (0.15)	1.30*** (0.02)
8. Alcoholic beverages	−1.43*** (0.05)	−0.01 (0.01)	−0.02** (0.01)	−0.03*** (0.01)	−0.04*** (0.01)	−0.16** (0.05)	1.55*** (0.02)
9. Chocolate and candy	0.01 (0.03)	−1.28*** (0.05)	0.01 (0.03)	−0.10** (0.04)	0.00 (0.04)	−0.06 (0.10)	1.13*** (0.01)
10. Desserts	−0.01 (0.02)	0.01 (0.03)	−1.20*** (0.03)	−0.02 (0.02)	−0.05* (0.02)	0.10 (0.07)	1.08*** (0.01)
11. Snacks	−0.04 (0.03)	−0.08* (0.03)	−0.01 (0.02)	−1.21*** (0.03)	0.04 (0.03)	0.20* (0.08)	0.97*** (0.01)
12. Fruit, vegetables, nuts and seeds	−0.01 (0.02)	0.01 (0.02)	−0.04* (0.02)	0.02 (0.02)	−1.05*** (0.03)	0.02 (0.05)	1.04*** (0.01)
13. Numeraire	0.07*** (0.01)	0.01 (0.00)	0.01*** (0.00)	0.01** (0.00)	0.01*** (0.00)	−0.99*** (0.02)	0.87*** (0.00)

*Note.* LS and HS are defined as up to and over 4 g of sugar per 100 ml, respectively. Bottled water includes LS flavored water. LS and HS soft drinks include carbonated soft drinks, juice drinks and fruit-flavored drinks. Dairy drinks include plant-based milk substitutes. Snacks include sweet and savory crackers All other foods and beverages (packaged and non-packaged) are included in the numeraire. *e_ij_* refers to the elasticity of group *i* w.r.t. the price of group *j*. Own-price elasticities in boldface and standard errors in parentheses.

* *p* < 0.05, **p < 0.01, ***p < 0.001.

The cross-price elasticities indicate substitution between groups. For instance, the positive cross-price elasticity between 100% fruit juice and other soft drinks indicates that fruit juice is a moderate substitute, meaning that a 10% increase in the price of soft drinks will cause consumers to increase demand for 100% fruit juice by 0.3%–1.4% (all else constant). Bottled water is also a moderate substitute for 100% fruit juices, as we found that a 10% increase in the price of fruit juices may result in a 1.2% increase in the demand for bottled water. The cross-price elasticity between desserts and FVNS, and alcohol and high-sugar soft drinks is −0.05, implying that these groups are weak complements. Other complements are snacks and chocolates and candy, desserts, and high-sugar soft drinks (each with a cross-price elasticity of −0.08), and water and alcoholic drinks (−0.16).

**Figure 1.**
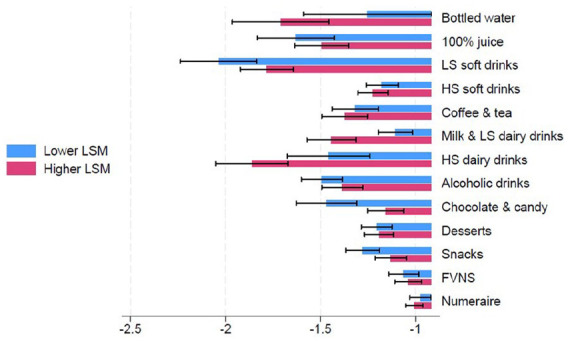
Price elasticities by LSM.

Given that different income groups respond to price changes differently, we stratified the analysis by LSM. Among lower LSM households (Figure 1, Supplemental Appendix B Table 3), the own-price elasticity for 100% fruit juice, low- and high-sugar soft drinks are −1.63, −2.04 and −1.18, respectively, while higher LSM households (Supplemental Appendix B Table 4) had own price elasticities of − 1.49, −1.78, and −1.22, respectively. This means that lower LSM households are more sensitive to the price of 100% fruit juice and low-sugar soft drinks compared to higher LSM households. Overall, higher LSM households are more sensitive to the price of bottled water, milk and dairy drinks, and slightly more sensitive to the price of high-sugar soft drinks, coffee and tea compared to lower LSM households. Meanwhile, lower LSM households were more sensitive to the price of 100% fruit juice, low-sugar soft drinks, alcohol, chocolate and candy and snacks than higher LSM households.

The total expenditure elasticities range from 0.97 for snacks to 1.55 for alcoholic drinks in the full sample. Comparing these elasticities between lower- and higher-LSM households, we found that a 1% increase in total expenditure on foods and beverages for at-home consumption increases the demand for each of the various food groups (holding all else constant) slightly more for lower-LSM households than for higher-LSM households (Supplemental Appendix B Tables 3 and 4).

## Discussion

Using detailed food and beverage purchase data among South African households from 2016 to 2019, we estimated total expenditure and uncompensated own- and cross-price elasticities of demand for eight beverage groups based on sugar content and four food groups that are generally high in sugar, sodium, saturated fats, or, conversely, seen as healthier. This is important considering the limited knowledge about the responsiveness of UPFs to price changes in South Africa. The estimated uncompensated own-price elasticities of demand are within the range found for similar food and beverage groups in countries at similar economic development levels and time periods such as Brazil, Chile and Indonesia.^27–29^ Our findings of −1.2 and −1.91 own-price elasticities of demand for high- and low-sugar soft drinks, respectively, also align with findings from a meta-analysis of 62 studies that found implied price elasticities of demand of −1.59.^30^ However, the own-price elasticities for low-sugar dairy drinks (−1.91) is higher than those reported in Mexico and Chile, ranging between −1.1 and −1.6.21.^31^ Meanwhile, lower price elasticities for packaged fruits, vegetables, nuts and seeds (−1.05) and for the numeraire (−0.99) compared to the other groups is reasonable as the numeraire contains staples and culinary ingredients. The cross-price elasticities were generally small, indicating weak to moderate complementarity or substitutability. There were a few exceptions as seen by the substitutability of low-sugar soft drinks and 100% fruit juices for high-sugar soft drinks, the substitutability of bottled water for low-sugar soft drinks, chocolate and candy, and the complementarity between bottled water and alcohol. Generally, the cross-price elasticities we found are smaller than those reported in other studies.^21,31^

The higher price responsiveness among lower-LSM households compared to higher-LSM households for all the food groups and some beverage groups is consistent with expectations. Interestingly, we found that higher LSM households were more responsive to the prices of bottled water and milk and dairy drinks than lower-LSM households. For bottled water, this may be because higher-LSM households are more likely to have good substitutes in the form of private potable water sources, particularly because the data used included a period of water crises in certain parts of the country.^32,33^ Meanwhile, lower-LSM households may be more reliant on milk and dairy products as sources of protein compared to higher-LSM households.^34^ There is a need to better understand the dietary patterns across various socio-demographic groups in South Africa from more recent years. Findings from the 2022 National Dietary Intake Survey^35^ and the 2022/23 Income and Expenditure Survey^36^ that are expected to be publicly available in due course could prove instructive.

Previous price elasticity of demand estimates for South Africa, based on the 2010/11 Income and Expenditure Survey and Consumer Price Index microdata, are consistent with ours for comparable food, and beverage groups. One study found own-price elasticity estimates of −1.78 and −0.99 for dairy and fruits and vegetables, respectively, similar to what we found for milk and dairy drinks (−1.25–−1.67) and FVNS (−1.05)0.22 Another found estimates of −1.1 for milk, −1.18 for carbonated soft drinks, and −0.44 for fruit juices, compared to −1.25 for milk (and low-sugar dairy drinks), −1.2 for high-sugar soft drinks, and −1.55 for 100% juice (which excludes other juice drinks).^20^

These previous studies used broad food and beverage groups. In using household-level and more disaggregate information on food purchases down to the item level, we can classify packaged products into nutritionally meaningful groups based on sugar content for beverages. Similarly, we focused on food groups that are generally considered healthier, or that tend to be high in sugar, sodium and or saturated fats based on the nutrient-profiling model of the proposed regulation R3337 for Food Labeling in South Africa.^8,9^

Our findings can be used to inform on fiscal policy options that South Africa’s National Treasury might consider, including expanding the HPL to cover other high sugar, sodium or saturated fat products. For example, our findings suggest that if the HPL was expanded to include 100% juice, an excise tax equivalent to a 10% price increase could result in a reduction in purchases of this sugary drink by 15.5%. If the HPL was expanded to include sweetened drinks containing non- sugar sweeteners, an excise tax equivalent to a 10% price increase could result in a reduction in purchases of such drinks by 19%. Moreover, the cross-price elasticities show that increasing the prices of all sweet drinks (100% juice, low-sugar soft drinks, high-sugar soft drinks and high-sugar dairy drinks) will result in an increase in the demand for bottled water. More importantly, our findings show the feasibility of extending the HPL to other sugary drinks and UPFs.

In considering the cross-price elasticities, it is also clear that if the HPL was expanded to include these other sweet drinks, there is a need to also raise the current HPL excise tax on SSBs to mitigate substitutions to SSBs. The results also prove it feasible to raise the HPL. Given that at its introduction, the HPL had an effective tax burden of approximately 10% of the per-liter price of the most popular SSB,19 which is substantially below the 20% minimum threshold recommended by the World Health Organization,^37^ it is important that the government raises the HPL and extend it to all sweet drinks. From a fiscal perspective, increasing the HPL and expanding it to all sweet drinks will help the government raise additional revenue for health expenditures and other fiscal requirements. In addition, regardless of whether the tax is expanded, the HPL should be adjusted yearly for inflation to preserve incentives for consumers to substitute high-sugar beverages with lower-sugar beverages and for producers to reduce sugar, or at minimum to preserve real tax revenue. While many sugary drinks taxes do not apply to 100% juice, as of October 2024, Thailand does not have such exemptions from its sugar-based volumetric excise tax on beverages. Malaysia, Nepal and Bangladesh also include 100% juices in their tax base. Regarding unhealthy foods, several countries have implemented taxes on such foods, including but not limited to Hungary (packaged foods high in salt, sugar, or saturated fat), Mexico (non-essential energy dense foods), Ethiopia (products high in saturated or hydrogenated fats, certain sugars, and confectionery), and most recently Colombia (ultra-processed foods high in free sugars, sodium, and saturated fats). Evaluations of Hungary and Mexico’s taxes have shown that they were associated with price increases and purchase decreases for the taxed foods, although in the case of Hungary effects on consumer spending on those foods did not persist past the first year, amidst an economic upturn. This means it is possible for South Africa to extend the tax to cover all food and beverages containing high levels of “nutrients of concern.”

Our findings also suggest that lower-LSM South Africans are generally more price sensitive than higher-LSM households for food and beverage groups that are most likely to have many products that will require warning labels under the proposed Regulation 3337.^38^ Products that will require warning labels include all packaged foods containing high levels of “nutrients of concern,” such as sugar, unhealthy fats, salt, and artificial sweeteners. Current research shows that low-income adults in South Africa consume large amounts of UPF.6 The consumption of these foods has been shown to increase the risk of NCDs in the South African population.^10^ This means that if the HPL is expanded to these food or beverage groups, lower-LSM households will reduce their purchases of such products more relative to higher-LSM households. Importantly, this also means that a substantial share of the tax revenue may be paid by higher-LSM households.

Future work will use these estimates to simulate changes in purchases by LSM group under different fiscal policy scenarios including taxes and subsidies, and from that determine the population and subpopulation-specific nutritional implications and estimate the potential tax revenues that might be generated.

## Limitations

We note some limitations of this work. First, the data used does not include the lowest income households in South Africa (LSM 1–3). However, the data does represent 95% of total household population,^7^ and is stratified by two LSM groups to deepen understanding of the potential implications of price changes for a large share of the population. Second, the data reflects pre-Covid-19 demand which could have changed over time; nonetheless this reflects the most recent estimates available. Relatedly, the data is somewhat dated, but by no means overly so compared to other studies of consumer demand using similar datasets.^24–26^ A final limitation is that with budget shares as the dependent variables, our elasticity estimates represent the total response to price changes on both the quantity and quality margins and should therefore be viewed as upper bound estimates of elasticities of quantity.^39^ The data did not allow for the construction of satisfactory Laspeyres group price indices to use in the joint estimation of budget share and unit value equations to account for the effect of prices on quality choices, nor for Deaton’s spatial estimation approach^40^—if willing to impose weak separability—due to coarse geographical information.

## Conclusions

We found that a 10% rise in prices reduces the consumption of ultra-processed commodities by 10.5%–19.1%. Importantly, low-income South Africans are generally more price sensitive than high-income households for most of the unhealthy beverage/food groups. We also found that some goods are substitutes, for example, 100% fruit juice and other soft drinks, while beverage/food groups such as desserts, and alcohol and high-sugar soft drinks; and snacks and chocolates and candy, desserts are weak complements. These findings indicate that increasing the HPL and/or expanding it to all sweet drinks and unhealthy food groups (as defined in the proposed Regulation 3337) can help slow down and prevent the rising prevalence of obesity and obesity- related diseases. From a fiscal perspective, increasing the HPL and expanding it to all sweet drinks and other unhealthy foods will help the South African government raise additional revenue for health expenditures and other fiscal requirements.

## Supplemental Material

sj-docx-1-phj-10.1177_22799036251350956 – Supplemental material for Estimating price and expenditure elasticities for select foods and drinks in South Africa using a demand systems model Supplemental material, sj-docx-1-phj-10.1177_22799036251350956 for Estimating price and expenditure elasticities for select foods and drinks in South Africa using a demand systems model by Chengetai Dare, Maxime Bercholz, Micheal Kofi Boachie, Evelyn Thsehla and Shu Wen Ng in Journal of Public Health Research
