# Supplementary material for: Estimating price and expenditure elasticities for select foods and drinks in South Africa using a demand systems model
Source: J Public Health Res. 2025 Jun 29;14(2):22799036251350956. doi: 10.1177/22799036251350956 (PMC12206998; doi:10.1177/22799036251350956)
Supplement: sj-docx-1-phj-10.1177_22799036251350956 – Supplemental material for Estimating price and expenditure elasticities for select foods and drinks in South Africa using a demand systems model [file sj-docx-1-phj-10.1177_22799036251350956.docx]

# Supplemental Material

# Appendix A

## Model and elasticities

We used the modified EASI model of Zhen et al.,^1^ conditioning on total food and beverage expenditure for at home consumption due to the lack of income data. The model takes the following form:

$$\begin{aligned} w_{i,h,t}=\max\left( 0,w_{i,h,t}^{*} \right), w_{i,h,t}^{*}=\sum_{g=1}^{G} a_{i,g}{\ln p}_{g,h,t}+\sum_{r=1}^{L} b_{i,r}y_{h,t}^{r}+z_{h,t}^{'}c_{i}+u_{i,h,t}\#\left( 1 \right) \end{aligned}$$

where $i$, $h$, and $t$ index groups, households, and quarters, $w_{i,h,t}$ and $w_{i,h,t}^{*}$ are the observed and latent budget shares, $p_{g,h,t}$ is the price of group $g$ with $G=13$ the number of groups, $y_{h,t}$ is total food and beverage real expenditure for at-home consumption (henceforth, “total expenditure”), $z_{h,t}$ is a vector of demographics, $a_{i}=\left( a_{i,1},\ldots,a_{i,13} \right)^{'}$, $b_{i}=\left( b_{i,1},\ldots,b_{i,L} \right)^{'}$, and $c_{i}$ are vectors of parameters to be estimated with $L$ empirically determined, and $u_{i,h,t}$ is an IID error term. We used the linear approximation of this modified EASI model with $y_{h,t}=\ln x_{h,t}-\sum_{g=1}^{G} \bar{w}_{g}\ln p_{g,h,t}$, where $\bar{w}_{g}$ is the sample average budget share for group $g$. The demographics included: household size, family life cycle (empty nesters, families, mature without children, retired, and young without children), lower or higher LSM, and province and quarter fixed effects.

The $G\times1$ vector of total expenditure elasticities of demand for household $h$ in quarter $t$ (omitting the $h$ and $t$ subscripts for brevity) is given by:

$$\begin{aligned} e=W^{-1}\left\{ \left( I_{G}+bp^{'} \right)^{-1}b \right\}+ı_{G}\#\left( 2 \right) \end{aligned}$$

where $W$ is the $G\times G$ matrix with the observed budget shares in the diagonal and zeros off the diagonal, $I_{G}$ is the $G\times G$ identity matrix, $b$ is the $G\times1$ vector whose ith element is $\sum_{r=1}^{L} rb_{i,r}y^{r-1}$, $p$ is the $G\times1$ vector of log prices, and $ı_{G}$ is a $G\times1$ vector of ones.

The uncompensated price elasticity of group $i$ with respect to the price of group $j$ is given by:

$$\begin{aligned} e_{i,j}=h_{i,j}-w_{j}e_{i}\#\left( 3 \right) \end{aligned}$$

where $h_{i,j}=a_{i,j}w_{i}^{-1}+w_{j}-\delta_{ij}$ is the compensated price elasticity with $\delta_{i,j}=1$ for $i=j$ and $0$ otherwise, and $e_{i}$ is the total expenditure elasticity of group $i$.

One difficulty with these elasticities is that they are undefined when $w_{i}=0$ for any group $i$. Following Zhen et al.,^1^ we used the expected budget shares in place of the observed and the estimated marginal effects of the log prices and total expenditure terms on the expected budget shares in place of the $a_{i,j}$ and $b_{i,r}$ coefficients in equations 2 and 3. The elasticities in the text are sample averages across all household-quarters, weighted by the household weights.

## Prices

Unlike individual items, groups of goods do not have a natural definition of price. A simple but naïve approach is to use unit values, i.e. the ratio of total group expenditure to total group quantity. This is problematic on several counts.^2, 3^ First, unit values reflect the dual quantity and quality choices made by the consumer. As a result, unit values are simultaneously determined with the dependent variables, be they quantities or—because quality choices affect expenditure—budget shares. Furthermore, unobserved household heterogeneity may aggravate endogeneity.^1^ Second, the ability of consumers to adjust both quantity and quality in response to price changes implies that we should expect price elasticities of unit values to be less than one. As a result, the *unit value* elasticities will typically exaggerate the *price* elasticities, with any change in demand effectively attributed to a smaller price change than has actually occurred. For example, if a 10% increase in meat prices leads to a 5% increase in the unit value of meat and a 5% decrease in the demand for meat, interpreting the unit value elasticity as a price elasticity would lead us to conclude that the demand for meat has a negative unit price elasticity, double its true value of -.5. Third, measurement errors in expenditures and quantities, if present, will lead to measurement errors in unit values.

Borrowing from previous applications,^1, 4^ we constructed household-quarter Fisher price indices for the food and beverage groups (excluding the numeraire), with group-brand elements and base prices and quantities set at their sample means. That is, we defined the price of group $g$ for household $h$ in quarter $t$ as

$$\begin{aligned} p_{g,h,t}=\sqrt{\frac{\sum_{k} v_{k,h,t}q_{k}^{0}}{\sum_{k} v_{k}^{0}q_{k}^{0}}*\frac{\sum_{k} v_{k,h,t}q_{k,h,t}}{\sum_{k} v_{k}^{0}q_{k,h,t}}} \#\left( 4 \right) \end{aligned}$$

where $v_{k,h,t}=x_{k,h,t}/q_{k,h,t}$ and $q_{k,h,t}$ are the unit value and quantity purchased of element $k$ for household $h$ in quarter $t$, with $x_{k,h,t}$ household $h$’s total expenditure on element $k$ in quarter $t$, and $p_{k}^{0}$ and $q_{k}^{0}$ are element $k$’s base values.

We imputed missing unit values due to non-purchasing using predictions from group-level OLS regressions of observed unit values on province, quarter, brand, all pairwise interactions of province, quarter, and brand, individual LSM group, family life cycle, and log household size, replacing non-positive predictions by the smallest positive prediction. For these imputations, we split desserts by unit of measure, as it included both items in grams and items in milliliters. Appendix Table 2 reports the proportion of imputed missing values by group. In keeping with previous applications, we also set $q_{k,h,t}=1$ for all $k$ for household-quarters with zero group expenditure.

To reduce the number of imputations, we aggregated elements into private-label and non-private-label composites (separately within each province) if their shares of weighted total group expenditure was less than .5%, and dropped observations for elements that were not purchased by at least one household in the quarter.

Numeraire prices were backed out using the quarterly CPI for foods and non-alcoholic beverages by solving $\ln CPI_{t}^{FNAB}=\sum_{g=1}^{G} {\bar{\tilde{w}}}_{g,t}\ln p_{g,h,t}$ for the numeraire price, $p_{G,h,t}$, where ${\bar{\tilde{w}}}_{g,t}$ is the sample average share of total food and non-alcoholic beverage expenditure for group $g$ in quarter $t$ (set to zero for alcoholic beverages).^1^ We obtained monthly CPI data from Statistics South Africa’s website and derived quarterly averages rebased by the study period average, in line with the Fisher price indices. The sample mean shares were used in place of household-level shares because low numeraire shares resulted in some extremely high index values. Appendix Figure 1 plots the mean and median index values over time by group.

## Estimation

Although Fisher price indices help to address price endogeneity due to the dual nature of quantity and quality choices,^1^ unobserved household heterogeneity and measurement error may still be a concern. To mitigate these issues, in particular unobserved household heterogeneity, we followed Valizadeh and Ng^40^ and augmented the model with correlated random effects by adding the within-household means of the log prices, leveraging the sample’s longitudinal design.

We followed their estimation procedure, which consists in estimating all equations excluding the numeraire separately by maximum likelihood and deriving estimates of the parameters and variance-covariance matrix for the system, with the theoretical restrictions imposed. In what follows, $d$ is the number of parameters per equation including the error variance (number of explanatory variables including the intercept, plus one), $m=G-1$ is the number of equations excluding the numeraire, and $N$ is the number of observations.

The free parameters (as opposed to those derived from the theoretical restrictions, e.g., one of $a_{1,2}$ and $a_{2,1}$, which are constrained to be equal by symmetry) and variance-covariance matrix for the system composed of the first $m$ equations are respectively estimated as^40^ $\left( R^{'}C^{-1}R \right)^{-1}R^{'}C^{-1}\hat{\theta}$ and $N^{-1}\left( R^{'}C^{-1}R \right)^{-1}$, where:

- $R$ is the $dm\times f\left( d,m \right)$ matrix imposing the theoretical restrictions of homogeneity and symmetry with $f\left( d,m \right)$ the number of free parameters and $f$ a function of the restrictions, defined such that $\theta_{i}=\sum_{j} r^{i,j}\theta_{j}$ with $\theta_{k}$ the $k$th parameter and $r^{i,j}$ the ith row, jth column entry of $R$
- $C$ is the $dm\times dm$ matrix defined as $C=G^{-1}\Psi{G^{-1}}^{'}$ with

$$\underset{dm\times dm}{\underbrace{G}}=N^{-1}\left( \begin{matrix} \hat{\Sigma}_{1}^{-1} & \cdots& 0 \\ \vdots& \ddots& \vdots\\ 0 & \cdots& \hat{\Sigma}_{m}^{-1} \end{matrix} \right)$$

$$\underset{dm\times dm}{\underbrace{\Psi}}=N^{-1}\left( \begin{matrix} J_{1}^{'}J_{1} & \cdots& J_{1}^{'}J_{m} \\ \vdots& \ddots& \vdots\\ J_{m}^{'}J_{1} & \cdots& J_{m}^{'}J_{m} \end{matrix} \right)$$

where $\hat{\Sigma}_{i}$ is the $d\times d$ single-equation tobit estimate of the variance-covariance matrix for equation $i$ and $J_{i}$ is the $N\times d$ Jacobian of the observation-level log-likelihoods for equation $i$ with respect to the parameters, evaluated at the tobit estimates,

$$J_{i}=\left. \left( \begin{matrix} \frac{\partial l_{i,1}}{\partial\theta_{i,1}} & \cdots& \frac{\partial l_{i,1}}{\partial\theta_{i,d}} \\ \vdots& \ddots& \vdots\\ \frac{\partial l_{i,N}}{\partial\theta_{i,1}} & \cdots& \frac{\partial l_{i,N}}{\partial\theta_{i,d}} \end{matrix} \right) \right|_{\theta_{i}=\hat{\theta}_{\boldsymbol{i}}}$$

- and $\hat{\theta}$ is the $dm\times1$ vector of the single-equation tobit estimates:

$$\hat{\theta}=\left( \hat{\theta}_{1,1},\hat{\theta}_{1,d},\ldots,\hat{\theta}_{m,1},\hat{\theta}_{m,d} \right)^{'}$$

The theoretical restrictions of homogeneity and symmetry imply $d-1$ free parameters for the first equation (dropping $a_{1,G}$ due to homogeneity), $d-2$ free parameters for the second equation (dropping $a_{2,G}$ due to homogeneity and $a_{2,1}$ due to symmetry), down to $d-m$ free parameters for the $m$th equation (dropping $a_{m,G}$ due to homogeneity and $a_{m,1},\ldots,a_{m,m-1}$ due to symmetry), i.e. a total of $dm-m\left( m+1 \right)/2$free parameters.

$L$ was determined empirically by adding one more power and testing the joint significance of the additional power across the first $m$ equations, starting with $L=1$.^1^ This yielded $L=4$, which is what Zhen et al. and Valizadeh and Ng found on US data, too.^1, 4^

Lastly, we recovered the parameters of the numeraire equation using the adding-up restriction to calculate observation-level elasticities and their (sub)sample averages. The standard errors of the latter were obtained by simulation in two steps. In the first step, we drew 50 sets of random parameters from a multivariate normal distribution with means and covariances set at their estimated values. We then recalculated the average elasticity estimates for each set of random parameters and calculated the standard errors as the standard deviations over these 50 sets of estimates.

# Appendix B

Appendix Table 1: Weighted mean quarterly expenditure (*x*) and quantity (*q*) per person, and unit value (*v*) by group and living standards measure (LSM)

|  | Lower LSM | | | | | Higher LSM | | | | | All | | | | |
| --- | --- | --- | --- | --- | --- | --- | --- | --- | --- | --- | --- | --- | --- | --- | --- |
|  | *x* | *q* | *x*\|*x* > 0 | *q*\|*x* > 0 | *v*\|*x* > 0 | *x* | *q* | *x*\|*x* > 0 | *q*\|*x* > 0 | *v*\|*x* > 0 | *x* | *q* | *x*\|*x* > 0 | *q*\|*x* > 0 | *v*\|*x* > 0 |
| Bottled water | 5 | 1 | 22 | 2 | 11.5 | 7 | 1 | 25 | 4 | 10.2 | 6 | 1 | 23 | 3 | 10.9 |
| 100% juice | 15 | 1 | 41 | 2 | 23.2 | 23 | 1 | 48 | 3 | 22.0 | 18 | 1 | 45 | 2 | 22.7 |
| LS soft drinks | 8 | 2 | 30 | 8 | 8.4 | 14 | 3 | 41 | 8 | 8.7 | 10 | 2 | 35 | 8 | 8.6 |
| HS soft drinks | 86 | 11 | 100 | 13 | 8.6 | 77 | 10 | 91 | 12 | 9.2 | 82 | 11 | 97 | 13 | 8.8 |
| Coffee & tea | 47 | 31 | 66 | 44 | 1.9 | 61 | 32 | 84 | 44 | 2.5 | 53 | 32 | 73 | 44 | 2.1 |
| Milk & LS dairy drinks & alternatives | 49 | 11 | 64 | 14 | 7.7 | 56 | 8 | 73 | 11 | 9.7 | 52 | 10 | 68 | 13 | 8.5 |
| HS dairy drinks & alternatives | 6 | 1 | 28 | 2 | 16.8 | 10 | 1 | 35 | 3 | 15.8 | 8 | 1 | 31 | 3 | 16.3 |
| Alcoholic beverages | 117 | 4 | 361 | 11 | 54.3 | 94 | 2 | 284 | 7 | 64.5 | 108 | 3 | 329 | 9 | 58.5 |
| Chocolate & candy | 20 | 0 | 41 | 0 | 152.3 | 53 | 0 | 76 | 1 | 158.1 | 33 | 0 | 58 | 0 | 155.2 |
| Desserts | 39 | 1 | 55 | 2 | 50.9 | 57 | 1 | 72 | 2 | 56.7 | 46 | 1 | 63 | 2 | 53.5 |
| Snacks | 31 | 0 | 44 | 1 | 90.7 | 51 | 1 | 63 | 1 | 95.8 | 40 | 0 | 52 | 1 | 93.0 |
| Fruit, vegetables, nuts & seeds | 67 | 3 | 83 | 3 | 29.3 | 117 | 5 | 132 | 5 | 30.5 | 87 | 4 | 104 | 4 | 29.8 |
| Numeraire | 854 | . | 859 | . | . | 1,167 | . | 1,171 | . | . | 980 | . | 985 | . | . |

Notes: Expenditure is in Rands and quantities are in liters for beverages and ice creams (desserts) and kilograms for foods (assuming a unit density for custard, dairy-based desserts, and preserved vegetables).

Appendix Table 2: Imputed group-brand unit values (% of household-quarters)

| Plain water & LS flavored water | 98.81 |
| --- | --- |
| 100% juice | 98.10 |
| LS CSDs, juice & fruit-flavored drinks | 98.84 |
| HS CSDs, juice & fruit-flavored drinks | 94.11 |
| Coffee & tea | 95.80 |
| Milk & LS dairy drinks & alternatives | 97.22 |
| HS dairy drinks & alternatives | 99.07 |
| Alcoholic beverages | 98.79 |
| Chocolate & candy | 96.64 |
| Desserts in g (desserts other than ice cream) | 97.01 |
| Desserts in ml (ice cream) | 97.59 |
| Snacks | 95.50 |
| FVNS | 94.69 |
| Total | 96.96 |

Notes: LS and HS are defined as up to and over 4 g of sugar per 100 ml, respectively. Bottled water includes LS flavored water. LS and HS soft drinks include carbonated soft drinks, juice drinks and fruit-flavored drinks. Dairy drinks include plant-based milk substitutes. Snacks include sweet and savory crackers All other foods and beverages (packaged and non-packaged) are included in the numeraire.

Appendix Figure 1: Quarterly mean and median price index values by group


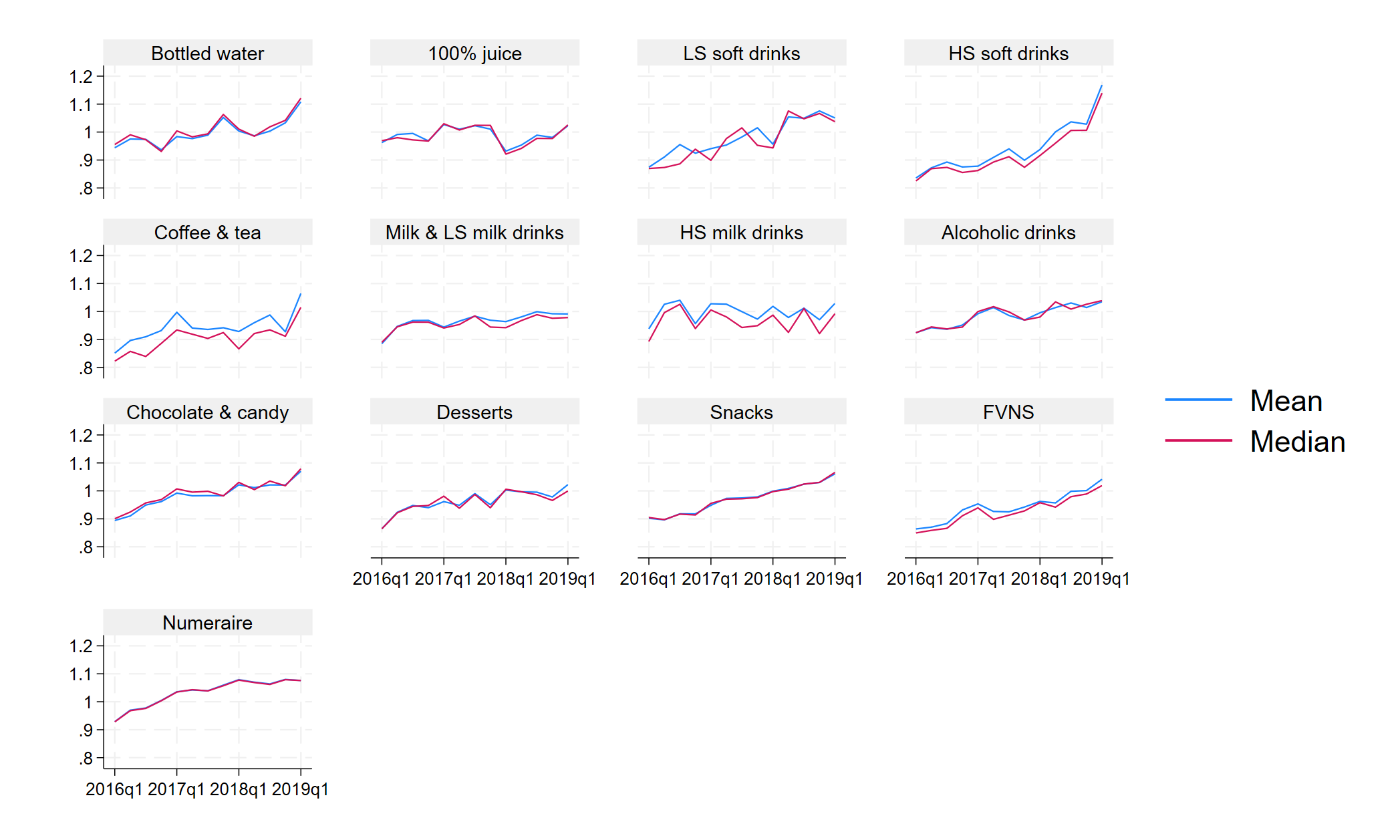


Appendix Table 3: Average price ($e_{i,j}$) and expenditure ($e_{i}$) elasticities, lower LSM

|  | $e_{i,j}$ | | | | | | |
| --- | --- | --- | --- | --- | --- | --- | --- |
|  | $j=1$ | $j=2$ | $j=3$ | $j=4$ | $j=5$ | $j=6$ | $j=7$ |
| 1. Bottled water | **-1.25***** | 0.15 | 0.09 | 0.20 | -0.17 | -0.01 | 0.07 |
|  | **(0.17)** | (0.12) | (0.11) | (0.15) | (0.09) | (0.12) | (0.12) |
| 2. 100% juice | 0.09 | **-1.63***** | 0.04 | 0.19** | -0.04 | 0.02 | 0.08 |
|  | (0.07) | **(0.10)** | (0.07) | (0.07) | (0.06) | (0.07) | (0.07) |
| 3. Low-sugar (LS) soft drinks | 0.07 | 0.05 | **-2.04***** | 0.15* | -0.00 | -0.08 | 0.08 |
|  | (0.08) | (0.10) | **(0.10)** | (0.07) | (0.09) | (0.07) | (0.08) |
| 4. High-sugar (HS) soft drinks | 0.05 | 0.08** | 0.05** | **-1.18***** | 0.04 | 0.01 | 0.04 |
|  | (0.03) | (0.03) | (0.02) | **(0.04)** | (0.03) | (0.03) | (0.03) |
| 5. Coffee & tea | -0.05 | -0.01 | 0.00 | 0.05 | **-1.32***** | 0.03 | 0.08* |
|  | (0.03) | (0.03) | (0.04) | (0.04) | **(0.06)** | (0.03) | (0.03) |
| 6. Milk & LS dairy drinks & alternatives | 0.00 | 0.02 | -0.03 | 0.02 | 0.04 | **-1.11***** | -0.01 |
|  | (0.04) | (0.04) | (0.03) | (0.04) | (0.03) | **(0.05)** | (0.04) |
| 7. HS dairy drinks & alternatives | 0.05 | 0.10 | 0.07 | 0.11 | 0.16* | -0.05 | **-1.46***** |
|  | (0.09) | (0.09) | (0.07) | (0.11) | (0.08) | (0.09) | **(0.11)** |
| 8. Alcoholic beverages | -0.02* | 0.01 | -0.02 | -0.07** | -0.03* | -0.03* | 0.04* |
|  | (0.01) | (0.02) | (0.01) | (0.02) | (0.01) | (0.01) | (0.01) |
| 9. Chocolate & candy | 0.16* | 0.07 | 0.03 | 0.05 | 0.12 | -0.08 | 0.03 |
|  | (0.07) | (0.07) | (0.05) | (0.07) | (0.06) | (0.06) | (0.06) |
| 10. Desserts | 0.00 | -0.02 | 0.03 | -0.07 | 0.08* | -0.00 | -0.01 |
|  | (0.03) | (0.04) | (0.04) | (0.04) | (0.04) | (0.03) | (0.03) |
| 11. Snacks | -0.04 | 0.06 | 0.02 | -0.02 | 0.08* | 0.02 | 0.04 |
|  | (0.05) | (0.05) | (0.04) | (0.04) | (0.04) | (0.04) | (0.04) |
| 12. Fruit, vegetables, nuts & seeds | 0.04 | -0.00 | -0.03 | -0.07 | 0.02 | -0.02 | -0.05 |
|  | (0.03) | (0.04) | (0.02) | (0.04) | (0.03) | (0.03) | (0.04) |
| 13. Numeraire | -0.01 | 0.00 | 0.02** | 0.01 | 0.01 | 0.01* | -0.01 |
|  | (0.01) | (0.01) | (0.01) | (0.01) | (0.01) | (0.01) | (0.01) |

Notes: * p < .05, ** p < .01, *** p < .001. LS and HS are defined as up to and over 4 g of sugar per 100 ml, respectively. Bottled water includes LS flavored water. LS and HS soft drinks include carbonated soft drinks, juice drinks and fruit-flavored drinks. Dairy drinks include plant-based milk substitutes. Snacks include sweet and savory crackers All other foods and beverages (packaged and non-packaged) are included in the numeraire. $e_{ij}$ refers to the elasticity of group $i$ w.r.t. the price of group $j$. Own-price elasticities in boldface and standard errors in parentheses.

Appendix Table 3 (continued): Average price ($e_{i,j}$) and expenditure ($e_{i}$) elasticities, lower LSM

|  | $e_{i,j}$ | | | | | | $e_{i}$ |
| --- | --- | --- | --- | --- | --- | --- | --- |
|  | $j=8$ | $j=9$ | $j=10$ | $j=11$ | $j=12$ | $j=13$ |  |
| 1. Bottled water | -0.14 | 0.27 | -0.00 | -0.09 | 0.09 | -0.67* | 1.32*** |
|  | (0.08) | (0.13) | (0.08) | (0.12) | (0.09) | (0.28) | (0.03) |
| 2. 100% juice | 0.05 | 0.06 | -0.04 | 0.07 | -0.02 | -0.32 | 1.29*** |
|  | (0.07) | (0.07) | (0.06) | (0.06) | (0.07) | (0.21) | (0.02) |
| 3. LS soft drinks | -0.12 | 0.04 | 0.04 | 0.02 | -0.09 | 0.37 | 1.34*** |
|  | (0.08) | (0.07) | (0.07) | (0.07) | (0.07) | (0.27) | (0.03) |
| 4. HS soft drinks | -0.04 | 0.02 | -0.03 | -0.01 | -0.04 | 0.06 | 0.96*** |
|  | (0.04) | (0.03) | (0.02) | (0.02) | (0.03) | (0.06) | (0.01) |
| 5. Coffee & tea | -0.00 | 0.07* | 0.06* | 0.06* | 0.02 | -0.09 | 1.06*** |
|  | (0.03) | (0.03) | (0.03) | (0.03) | (0.04) | (0.11) | (0.01) |
| 6. Milk & LS dairy drinks & alternatives | -0.00 | -0.04 | 0.00 | 0.01 | -0.02 | 0.13 | 0.99*** |
|  | (0.03) | (0.03) | (0.03) | (0.03) | (0.03) | (0.10) | (0.01) |
| 7. HS dairy drinks & alternatives | 0.21** | 0.03 | -0.03 | 0.06 | -0.13 | -0.63* | 1.34*** |
|  | (0.08) | (0.08) | (0.06) | (0.06) | (0.09) | (0.24) | (0.03) |
| 8. Alcoholic beverages | **-1.49***** | -0.01 | -0.02 | -0.04** | -0.04** | -0.16 | 1.59*** |
|  | **(0.06)** | (0.01) | (0.01) | (0.01) | (0.01) | (0.09) | (0.02) |
| 9. Chocolate & candy | -0.03 | **-1.47***** | -0.00 | -0.14** | -0.04 | 0.06 | 1.16*** |
|  | (0.05) | **(0.08)** | (0.05) | (0.05) | (0.06) | (0.16) | (0.02) |
| 10. Desserts | 0.00 | 0.00 | **-1.20***** | -0.06 | -0.01 | 0.09 | 1.11*** |
|  | (0.04) | (0.03) | **(0.04)** | (0.03) | (0.04) | (0.12) | (0.01) |
| 11. Snacks | -0.06 | -0.11** | -0.06 | **-1.28***** | 0.07 | 0.28* | 1.00*** |
|  | (0.04) | (0.04) | (0.04) | **(0.05)** | (0.05) | (0.11) | (0.01) |
| 12. Fruit, vegetables, nuts & seeds | -0.01 | -0.02 | -0.00 | 0.05 | **-1.06***** | 0.05 | 1.06*** |
|  | (0.03) | (0.03) | (0.03) | (0.03) | **(0.04)** | (0.09) | (0.01) |
| 13. Numeraire | 0.08*** | 0.01* | 0.02* | 0.02** | 0.02** | **-0.98***** | 0.86*** |
|  | (0.01) | (0.01) | (0.01) | (0.00) | (0.01) | **(0.03)** | (0.00) |

Notes: * p < .05, ** p < .01, *** p < .001. LS and HS are defined as up to and over 4 g of sugar per 100 ml, respectively. Bottled water includes LS flavored water. LS and HS soft drinks include carbonated soft drinks, juice drinks and fruit-flavored drinks. Dairy drinks include plant-based milk substitutes. Snacks include sweet and savory crackers All other foods and beverages (packaged and non-packaged) are included in the numeraire. $e_{ij}$ refers to the elasticity of group $i$ w.r.t. the price of group $j$. Own-price elasticities in boldface and standard errors in parentheses.

Appendix Table 4: Average price ($e_{i,j}$) and expenditure ($e_{i}$) elasticities, higher LSM

|  | $e_{i,j}$ | | | | | | |
| --- | --- | --- | --- | --- | --- | --- | --- |
|  | $j=1$ | $j=2$ | $j=3$ | $j=4$ | $j=5$ | $j=6$ | $j=7$ |
| 1. Bottled water | **-1.71***** | 0.11 | 0.19* | 0.08 | 0.07 | 0.03 | 0.09 |
|  | **(0.13)** | (0.09) | (0.09) | (0.10) | (0.06) | (0.09) | (0.10) |
| 2. 100% juice | 0.06 | **-1.49***** | 0.02 | 0.11* | 0.03 | 0.04 | 0.07 |
|  | (0.05) | **(0.07)** | (0.05) | (0.05) | (0.04) | (0.06) | (0.05) |
| 3. Low-sugar (LS) soft drinks | 0.14* | 0.03 | **-1.78***** | 0.16** | 0.01 | 0.03 | 0.08 |
|  | (0.07) | (0.08) | **(0.07)** | (0.06) | (0.07) | (0.06) | (0.07) |
| 4. High-sugar (HS) soft drinks | 0.02 | 0.06* | 0.06** | **-1.22***** | 0.03 | 0.01 | 0.00 |
|  | (0.02) | (0.02) | (0.02) | **(0.04)** | (0.03) | (0.03) | (0.03) |
| 5. Coffee & tea | 0.03 | 0.02 | 0.01 | 0.03 | **-1.37***** | 0.00 | 0.02 |
|  | (0.02) | (0.03) | (0.03) | (0.04) | **(0.06)** | (0.03) | (0.03) |
| 6. Milk & LS dairy drinks & alternatives | 0.02 | 0.03 | 0.02 | 0.01 | 0.01 | **-1.44***** | 0.05 |
|  | (0.04) | (0.04) | (0.03) | (0.04) | (0.03) | **(0.07)** | (0.05) |
| 7. HS dairy drinks & alternatives | 0.07 | 0.09 | 0.08 | -0.01 | 0.03 | 0.08 | **-1.86***** |
|  | (0.07) | (0.07) | (0.06) | (0.09) | (0.06) | (0.09) | **(0.10)** |
| 8. Alcoholic beverages | -0.03** | -0.02 | 0.00 | -0.03 | -0.01 | -0.01 | -0.01 |
|  | (0.01) | (0.01) | (0.01) | (0.02) | (0.01) | (0.01) | (0.01) |
| 9. Chocolate & candy | 0.08* | 0.03 | -0.00 | 0.05 | -0.01 | -0.03 | -0.00 |
|  | (0.04) | (0.04) | (0.04) | (0.04) | (0.04) | (0.04) | (0.04) |
| 10. Desserts | 0.02 | 0.00 | -0.00 | -0.07 | 0.09** | 0.00 | 0.04 |
|  | (0.02) | (0.03) | (0.03) | (0.04) | (0.03) | (0.03) | (0.03) |
| 11. Snacks | 0.05 | 0.08* | -0.06 | -0.05 | 0.02 | 0.01 | 0.07* |
|  | (0.04) | (0.04) | (0.03) | (0.03) | (0.03) | (0.04) | (0.03) |
| 12. Fruit, vegetables, nuts & seeds | -0.03 | 0.03 | 0.00 | 0.01 | -0.01 | 0.02 | -0.02 |
|  | (0.02) | (0.03) | (0.02) | (0.03) | (0.03) | (0.03) | (0.03) |
| 13. Numeraire | 0.00 | -0.00 | 0.01 | 0.01** | 0.01* | 0.02** | 0.01 |
|  | (0.00) | (0.01) | (0.01) | (0.00) | (0.01) | (0.01) | (0.01) |

Notes: * p < .05, ** p < .01, *** p < .001. LS and HS are defined as up to and over 4 g of sugar per 100 ml, respectively. Bottled water includes LS flavored water. LS and HS soft drinks include carbonated soft drinks, juice drinks and fruit-flavored drinks. Dairy drinks include plant-based milk substitutes. Snacks include sweet and savory crackers All other foods and beverages (packaged and non-packaged) are included in the numeraire. $e_{ij}$ refers to the elasticity of group $i$ w.r.t. the price of group $j$. Own-price elasticities in boldface and standard errors in parentheses.

Appendix Table 4 (continued): Average price ($e_{i,j}$) and expenditure ($e_{i}$) elasticities, higher LSM

|  | $e_{i,j}$ | | | | | | $e_{i}$ |
| --- | --- | --- | --- | --- | --- | --- | --- |
|  | $j=8$ | $j=9$ | $j=10$ | $j=11$ | $j=12$ | $j=13$ |  |
| 1. Bottled water | -0.17** | 0.18 | 0.04 | 0.10 | -0.10 | -0.26 | 1.25*** |
|  | (0.06) | (0.10) | (0.06) | (0.09) | (0.08) | (0.20) | (0.02) |
| 2. 100% juice | -0.04 | 0.04 | -0.01 | 0.09 | 0.04 | -0.29 | 1.22*** |
|  | (0.05) | (0.05) | (0.04) | (0.05) | (0.06) | (0.16) | (0.02) |
| 3. LS soft drinks | 0.04 | -0.01 | -0.01 | -0.11 | -0.01 | 0.06 | 1.27*** |
|  | (0.07) | (0.07) | (0.06) | (0.06) | (0.06) | (0.23) | (0.02) |
| 4. HS soft drinks | 0.01 | 0.04 | -0.04 | -0.03 | 0.02 | 0.14* | 0.93*** |
|  | (0.03) | (0.02) | (0.02) | (0.02) | (0.03) | (0.06) | (0.01) |
| 5. Coffee & tea | 0.03 | -0.00 | 0.09** | 0.01 | -0.01 | 0.06 | 1.05*** |
|  | (0.03) | (0.03) | (0.03) | (0.02) | (0.03) | (0.10) | (0.02) |
| 6. Milk & LS dairy drinks & alternatives | 0.03 | -0.02 | 0.00 | 0.01 | 0.03 | 0.27* | 0.99*** |
|  | (0.03) | (0.03) | (0.03) | (0.03) | (0.03) | (0.10) | (0.01) |
| 7. HS dairy drinks & alternatives | -0.05 | -0.01 | 0.06 | 0.11 | -0.06 | 0.07 | 1.27*** |
|  | (0.06) | (0.07) | (0.06) | (0.06) | (0.07) | (0.20) | (0.03) |
| 8. Alcoholic beverages | **-1.39***** | -0.00 | -0.03* | -0.03** | -0.04** | -0.16* | 1.53*** |
|  | **(0.05)** | (0.01) | (0.01) | (0.01) | (0.01) | (0.08) | (0.03) |
| 9. Chocolate & candy | 0.03 | **-1.16***** | 0.02 | -0.07* | 0.03 | -0.13 | 1.11*** |
|  | (0.03) | **(0.05)** | (0.03) | (0.03) | (0.04) | (0.10) | (0.01) |
| 10. Desserts | -0.03 | 0.02 | **-1.19***** | 0.01 | -0.08* | 0.12 | 1.05*** |
|  | (0.04) | (0.03) | **(0.04)** | (0.03) | (0.03) | (0.10) | (0.01) |
| 11. Snacks | -0.02 | -0.06* | 0.01 | **-1.13***** | 0.00 | 0.14 | 0.95*** |
|  | (0.03) | (0.03) | (0.03) | **(0.04)** | (0.04) | (0.09) | (0.01) |
| 12. Fruit, vegetables, nuts & seeds | -0.01 | 0.02 | -0.06* | -0.01 | **-1.04***** | -0.00 | 1.04*** |
|  | (0.03) | (0.03) | (0.02) | (0.03) | **(0.04)** | (0.08) | (0.02) |
| 13. Numeraire | 0.06*** | 0.00 | 0.01* | 0.01 | 0.01* | **-1.00***** | 0.88*** |
|  | (0.01) | (0.00) | (0.01) | (0.00) | (0.01) | **(0.02)** | (0.00) |

Notes: * p < .05, ** p < .01, *** p < .001. LS and HS are defined as up to and over 4 g of sugar per 100 ml, respectively. Bottled water includes LS flavored water. LS and HS soft drinks include carbonated soft drinks, juice drinks and fruit-flavored drinks. Dairy drinks include plant-based milk substitutes. Snacks include sweet and savory crackers All other foods and beverages (packaged and non-packaged) are included in the numeraire. $e_{ij}$ refers to the elasticity of group $i$ w.r.t. the price of group $j$. Own-price elasticities in boldface and standard errors in parentheses.

**References**

1. Zhen C, Finkelstein EA, Nonnemaker JM, et al. Predicting the effects of sugar‐sweetened beverage taxes on food and beverage demand in a large demand system. *American journal of agricultural economics* 2014; 96: 1-25.

2. Deaton A. Price Elasticities from Survey Data - Extensions and Indonesian Results. *J Econometrics* 1990; 44: 281-309. DOI: Doi 10.1016/0304-4076(90)90060-7.

3. Deaton A. Quality, Quantity, and Spatial Variation of Price. *Am Econ Rev* 1988; 78: 418-430.

4. Valizadeh P and Ng SW. Promoting Healthier Purchases: Ultraprocessed Food Taxes and Minimally Processed Foods Subsidies for the Low Income. *Am J Prev Med* 2024; 67: 3-14. 20240402. DOI: 10.1016/j.amepre.2024.02.019.
